# Tracking cortical entrainment in neural activity: auditory processes in human temporal cortex

**DOI:** 10.3389/fncom.2015.00005

**Published:** 2015-02-10

**Authors:** Andrew Thwaites, Ian Nimmo-Smith, Elisabeth Fonteneau, Roy D. Patterson, Paula Buttery, William D. Marslen-Wilson

**Affiliations:** ^1^Neurolex Group, Department of Psychology, University of CambridgeCambridge, UK; ^2^MRC Cognition and Brain Sciences UnitCambridge, UK; ^3^Department of Physiology, Development and Neuroscience, Centre for the Neural Basis of Hearing, University of CambridgeCambridge, UK; ^4^Department of Theoretical and Applied Linguistics, University of CambridgeCambridge, UK

**Keywords:** neural computation, magnetoencephalography, MNE source space, speech envelope, fundamental frequency contour, information encoding, model expression

## Abstract

A primary objective for cognitive neuroscience is to identify how features of the sensory environment are encoded in neural activity. Current auditory models of loudness perception can be used to make detailed predictions about the neural activity of the cortex as an individual listens to speech. We used two such models (loudness-sones and loudness-phons), varying in their psychophysiological realism, to predict the instantaneous loudness contours produced by 480 isolated words. These two sets of 480 contours were used to search for electrophysiological evidence of loudness processing in whole-brain recordings of electro- and magneto-encephalographic (EMEG) activity, recorded while subjects listened to the words. The technique identified a bilateral sequence of loudness processes, predicted by the more realistic loudness-sones model, that begin in auditory cortex at ~80 ms and subsequently reappear, tracking progressively down the superior temporal sulcus (STS) at lags from 230 to 330 ms. The technique was then extended to search for regions sensitive to the fundamental frequency (F0) of the voiced parts of the speech. It identified a bilateral F0 process in auditory cortex at a lag of ~90 ms, which was not followed by activity in STS. The results suggest that loudness information is being used to guide the analysis of the speech stream as it proceeds beyond auditory cortex down STS toward the temporal pole.

## Introduction

How features of the auditory environment are encoded in neural activity is of primary importance in speech perception. Questions regarding this encoding are tackled by hypothesizing models that specify which features of the environment are retained, and how they are represented in neural activity. In those situations where the environment changes over time, some of these features may be “tracked” by cortical current, a phenomenon known as cortical entrainment (Ding and Simon, 2014; Ding et al., 2014).

Recently, several groups have isolated speech-related activity in auditory cortex by correlating the envelopes of the speech waves with electro-encephalographic (EEG), magneto-encephalographic (MEG), and intracranial-EEG data, gathered while people were listening to speech (e.g., Ahissar et al., 2001; Luo and Poeppel, 2007; Aiken and Picton, 2008; Nourski et al., 2009; Kubanek et al., 2013). The assumption, either explicit or implicit, is that there is a region of the temporal lobe where the cortical current tracks, or is entrained by, the stimulus envelope, that is, the cortex calculates a neural version of the envelope which is temporally synchronized to the acoustic envelope. The associated regions of entrainment are often found to be located in, or around, auditory cortex, as would be expected.

There are also several studies showing cortical entrainment of the speech envelope after it has been convolved with an impulse response, estimated using the spectro-temporal response function (Aiken and Picton, 2008; Mesgarani et al., 2009; Ding and Simon, 2012; Pasley et al., 2012; Zion Golumbic et al., 2013) or evoked spread spectrum analysis (Lalor et al., 2009; Power et al., 2012). For more information on the interpretation of cortical entrainment by dynamic auditory features, see the review paper of Ding and Simon (2014).

In this paper, we test the hypothesis that models that are more psychophysiologically realistic might reveal stronger entrainment of cortical current. Specifically, we show that the internal, neural version of the speech envelope is closely related to a model referred to as “instantaneous-loudness-sones” by Glasberg and Moore (2002) in their dynamic model of loudness, which is the basis of the ANSI standard for loudness (ANSI S3.4-2007). We conduct a whole-brain search for entrainment of instantaneous-loudness-sones in the current of approximately 5000 sources across cortex, at a wide range of latencies (0–700 ms) estimated from EEG and MEG (EMEG) data of subjects listening to 480 isolated words. The current in each of these sources is estimated by applying a transform to the measurements of the EMEG sensor field. There are several techniques to derive an accurate transform (see Hauk et al., 2011 for comparison). In this study we use Minimum Norm Estimation (MNE) (Hämäläinen and Ilmoniemi, 1994), which characterizes sources as vertices distributed evenly over a triangular mesh representation of cortex.

The instantaneous-loudness-sones computation involves an auditory form of compression applied separately to the individual frequency channels created in the cochlea. For comparison, we also search for entrainment of a model similar to the acoustic speech envelope which does not include the channel-specific compression, and which Glasberg and Moore (2002) refer to as “instantaneous-loudness-phons.” The model that includes channel-specific compression shows much stronger cortical entrainment than the model without channel-specific compression, including regions beyond auditory cortex in the superior temporal sulcus.

Finally, we search for cortical entrainment at a specific spatiotemporal location to the fundamental frequency, F0, of the voiced segments of the speech (Tsanas et al., 2014). The F0 contours for the 400 words are derived with autocorrelation. The analysis reveals entrainment of current in a small region of auditory cortex.

## Materials and methods

We separate this part of the paper into four sections. The first two cover the general and specific assumptions we make regarding the three hypotheses. The third lays out the procedure we use in this study to search for cortical entrainment, and the fourth describes the materials and methods for the EMEG study on spoken words that provides the speech comprehension data analyzed here.

### Defining candidate models

The assumption of cortical entrainment imposes some trivial constraints on the models we can test. We can consider any model that takes a time varying signal as input and a time varying signal as output, with function *f*() characterizing the mechanism by which the information (in this case the speech stream) is transformed before it becomes cortically entrained. Thus, if both input *x*_1_, …, *x*_*m*_ and output *y*_1_, …, *y*_*n*_ are of duration *t*, the model takes the form:

(1) $$f\left(\left(x_{1},\;x_{2},\;x_{3},\text{ …},\;x_{t}\right)\right)\;=\;\left(y_{1},\;y_{2},\;y_{3},\text{ …},\;y_{t}\right)$$

where *f*() is bounded both by a set of formal requirements (Davis et al., 1994) and a requirement that *y*_*i*_ cannot be dependent on any *x*_*k*_ where *k* > *i* (this last requirement avoids hypothesizing a non-causal *f*() where a region can express an output before it has the appropriate input).

In the following section, we specify three candidate models, drawn from the domain of speech analysis, whose neural distribution we will seek to determine.

### Models for instantaneous loudness and F0

To explore the neural substrates for speech comprehension, we select two well-studied properties of speech—loudness and F0—explored in the context of a large set (480) of naturally spoken words and phrases. Both are salient properties of the auditory experience, and models for both have been found to entrain in cortical current (envelope-based: Ahissar et al., 2001; Aiken and Picton, 2008; Nourski et al., 2009; Kubanek et al., 2013; frequency-based: Henry and Obleser, 2012).

Both of the candidate models generate discrete time series that specify the value of a summary statistic computed from the sound wave at 1-ms intervals. In the case of loudness, the statistic is an estimate of the loudness evoked by the word for each such temporal sample (termed “instantaneous loudness” in the psychoacoustic literature). In the case of fundamental frequency, the statistic is a measure of the glottal period of the speech wave at each temporal sample. The operations that we use to characterize the model represent hypotheses about the processing that is applied to speech sounds as they pass up the auditory pathway, culminating in auditory cortex.

One such hypothesis is that, broadly speaking, the *auditory* calculation of loudness and F0 takes place in five stages (Moore et al., 1997; Glasberg and Moore, 2002):

1. The sound is passed through a fixed bandpass filter representing the transfer function of the outer and middle ear. This filter attenuates frequency components below 500 Hz and above 5000 Hz, and accentuates components around 3000 Hz.
2. The cochlea analyses the sound into a large number of overlapping frequency bands each of which has a width of about 12% of the center frequency of the channel. During the filtering process, the amplitude of the activity within each channel is strongly compressed (Irino and Patterson, 2006). This initial spectral analysis is common to the calculation of loudness and F0 in the auditory system.
3. In the case of loudness, the auditory system computes a running estimate of the level of activity in each channel. For F0, the auditory system computes a running estimate of the dominant period of the wave in each channel—a statistic that can be simulated with autocorrelation.
4. Averages of the loudness and F0 values across channels, spectrally weighted in the case of F0, are computed to produce summary loudness and F0 estimates for each successive 1-ms sampling period.
5. In auditory research, these sequences of momentary F0 and loudness values are time averaged to predict the perceived loudness or F0 properties of the sound sequence as a whole.

**Figure 1:**
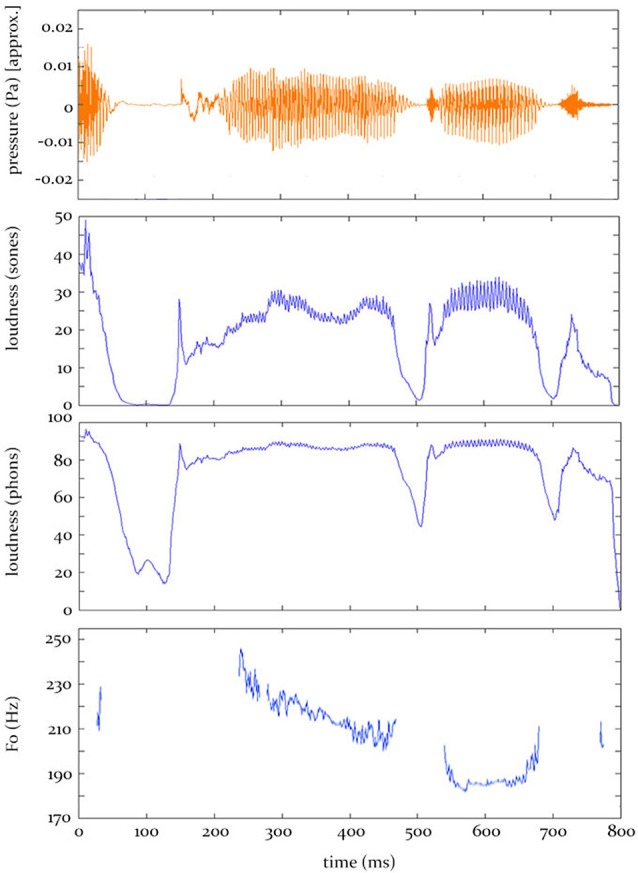
**Hypothesis predictions. Top:** The sound pressure contour for the word “applauded.” **Bottom:** The predicted electrophysiological activity, at some source, over time, given by the three models tested in this study.

In the current research, which does not address this fifth-stage of auditory *perception*, we concatenate the 1-ms model outputs from stage 4 across the entire word to generate time-varying contours of instantaneous loudness and F0. It is these predicted contours (see Figure 1) that are entered into the analysis procedure we use here (see Section “The Analysis Procedure” below).

#### The models for instantaneous loudness

A standard model for loudness (Moore et al., 1997) is designed to reflect the neurophysiological transformations of the auditory signal in the human ear and brain that give rise to the psychophysically measured model relating loudness (expressed in *sones*) to sound pressure level (in dB SPL) (e.g., Scharf, 1978). The function we employ here is designed to account for time-varying sounds (Glasberg and Moore, 2002) using an equation of the following form:

(2) $$\begin{array}{l} \text{loudness-sones}\left(x,\;t\right)\\ \;=\Delta_{ERB}\sum_{f}\text{compression}(\text{excitation}(x,t,f)) \end{array}$$

Following application of a filter that models transmission through the outer and middle ear, a short-term power spectrum *stps* of the waveform is assembled from six parallel fast fourier transforms with channel-specific Hanning window lengths that range from 2 to 64 ms. Spectral components that fall below -30 dB are omitted. Specific non-overlapping frequency ranges of spectral magnitudes are derived from each FFT. Thus components of *stps* in the highest frequency ranges (4050–15,000 Hz) are calculated with the 2 ms window, while those from the lowest range (20–80 Hz) are derived with the 64 ms window, and similarly for intermediate frequencies. As detailed in Moore et al. (1997), the components of the excitation pattern are the powers of the outputs of a bank of rounded-exponential filters (Patterson and Nimmo-Smith, 1980; Patterson et al., 1982). These can be calculated from *stps*, taking account of changes in auditory filter shape with center frequency and level. Here we use center frequencies *f* spaced at 0.25 ERB intervals. This yields a composite excitation pattern, excitation(*x*,*t*,*f*). Instantaneous loudness (loudness-sones) is derived as the area under the excitation pattern using a nonlinear compressive function (Moore et al., 1997), compression(), that emulates the compression that is known to occur in the cochlea. The window moves forward 1 ms at a time and the FFTs, excitation pattern and loudness summation are correspondingly updated.

A model satisfying (Equation 2), which we refer to as *loudness-sones*, is evaluated at 1-ms intervals to generate a sequence of momentary loudness estimates. Concatenated over the entire word, this sequence provides a prediction of the neural representation of the loudness contour of the word.

We contrast this prediction of the loudness contour with the contour generated by the output of a second model, termed *loudness-phons*. This is formed by converting the loudness-sones value into an external, acoustic-domain value (*phons*), which is defined as the level in dB SPL of a binaurally presented 1000-Hz tone that sounds equally loud to the sound in question (Stevens, 1936). The conversion from sones to phons reverses much of the channel specific cochlear compression applied at the auditory filtering stage in the sones calculation, making the phons contour similar to the log of the energy derived from the speech wave by full-wave rectification and temporal smoothing. Representations of the speech envelope generated along these lines are widely used in auditory neuroscience contexts.

#### The model for instantaneous F0

The vowels of speech are produced by the larynx in conjunction with the vocal tract: The vocal folds in the larynx produce a temporally regular stream of glottal pulses and the glottal period determines the fundamental frequency that we hear. The pulses subsequently excite resonances in the vocal tract which determine the vowel that we perceive. A good estimate of the glottal period in speech sounds is provided by the dominant lag in the autocorrelation of the wave (Licklider, 1956; Meddis and Hewitt, 1991). In the auditory system, in the second stage of processing, a coincidence mechanism that can be simulated with autocorrelation extracts an estimate of the glottal period as it occurs in each frequency channel. Then in the fourth stage, the individual period estimates are averaged to produce the momentary F0 estimate. This auditory F0 model can be expressed by:

(3) $$F0(x,t)=\sum_{a=1}^{nchan}\frac{\text{acpf}(\text{G}(a,\;c,\;x,\;t),w)}{nchan}$$

where G(*a*,*c*,*x*,*t*) is the output of a gammatone filterbank with *nchan* channels equally spaced on the ERB-rate scale (Hummersone et al., 2011) applied to the stimulus *x*, *a* is the channel number and *c* is a constant that determines the degree of compression applied to the channel output. acpf() is the peak *frequency* (1/lag) of the short term autocorrelation applied to the output of the current filter channel (ignoring the trivial peak at lag 0), and *w* is the window size over the autocorrelation. In the situation where no pitch period is identified for a channel, it is omitted from the averaging, with the denominator adjusted accordingly. In this paper, we test an F0 model satisfying (Equation 3) using the implementation based on that of Rabiner and Schafer (2010) (specifically Rabiner et al., 2014), where *w* is 20 ms and *nchan* is of size 32. The center frequencies of the highest and lowest bands in the filterbank were 6000 and 20 Hz, respectively. The window moves forward a millisecond at a time. The output of this model is a contour that follows the fundamental frequency of the speech sound, in Hertz. The value of constant *c* has no effect on the F0, and thus we do not compare F0 models with and without this compression, as we do for loudness.

The model also estimates the degree of periodicity (also known as Harmonic-to-Noise-Ratio and pitch-strength); defined as the ratio of the magnitude of the autocorrelation peak relative to the value at lag zero. When this periodicity value drops below 35%, the model makes no predictions (characterized as NaN values IEEE Computer Society, 2008) of how the neural signal will vary, and these intervals are subsequently ignored during the analysis (see Figure 1). As such, the F0 contour and pitch-strength contour share little variance in this dataset (16%). The F0 and loudness-sones contours share 33% variance, while the loudness-sones and loudness-phons contours share 70% variance.

### The analysis procedure

**Figure 2:**
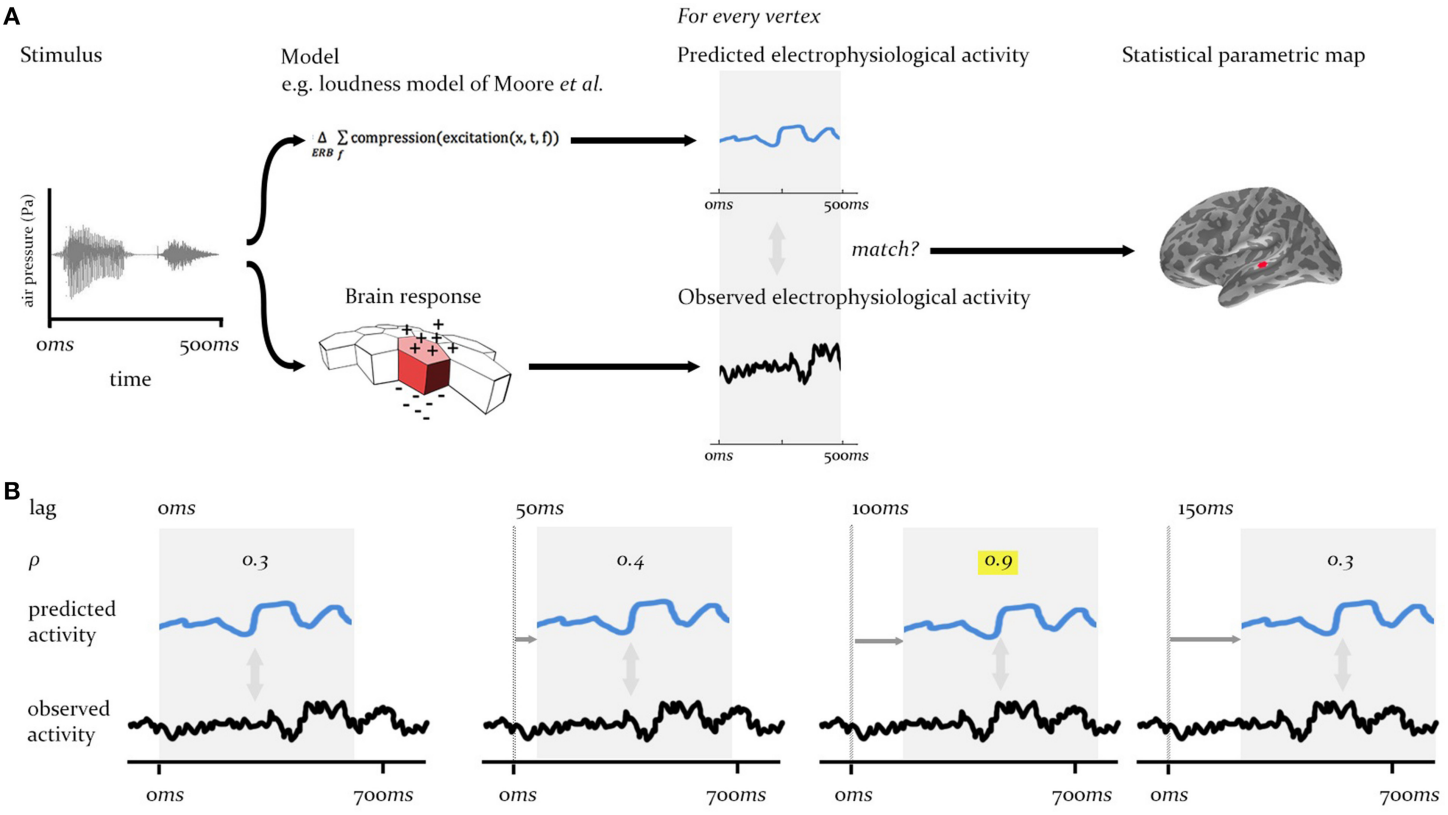
**Technique overview**. First **(A)**, the electrophysiological activity of the brain in response to a given stimulus (measured using EMEG) is matched to the pattern of neural activity predicted by the model being evaluated. Predicted and observed activity are tested for similarity (see Figure 3A) and the resulting SPM displays the regions (vertices) where the match is statistically significant. Second **(B)**, this procedure is repeated at different lags (illustrated here from 0 to 150 ms) between the onset of the observed neural activity and the onset of the predicted output. The similarity (Pearson's ρ) will be highest at the correct lag (highlighted). This produces an SPM that changes over time.

**Figure 3:**
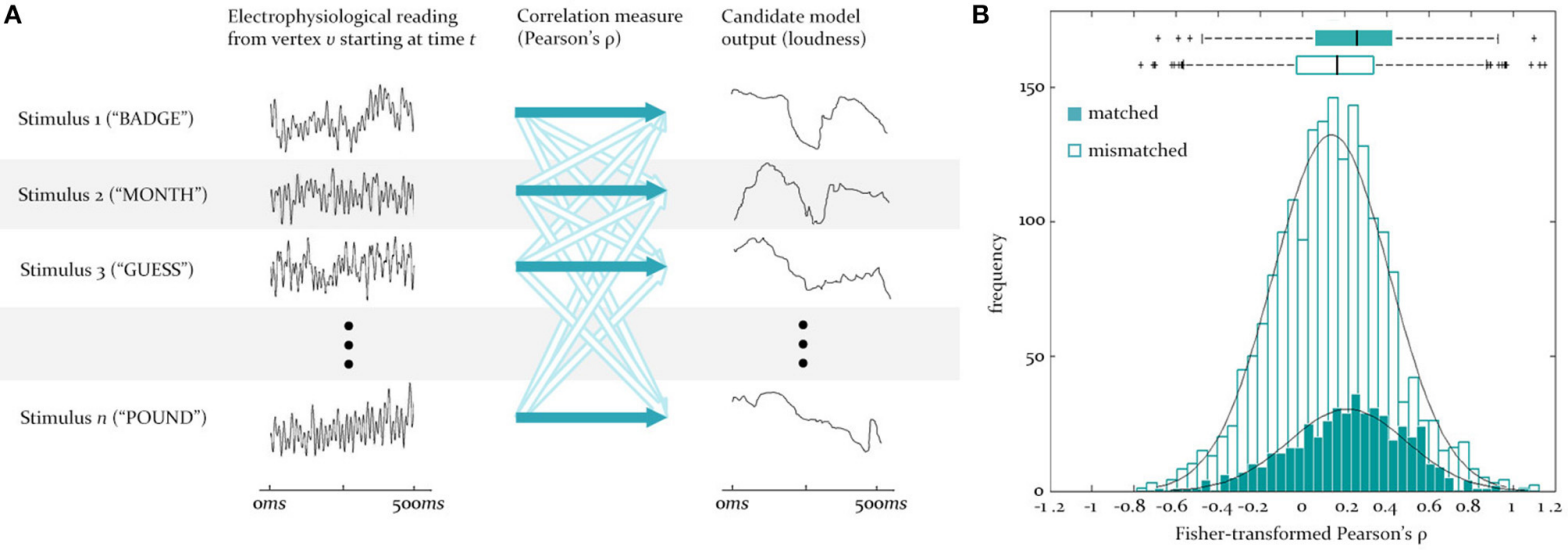
**Testing for similarity between source signals and model outputs**. **(A)** To test for the similarity between the predicted and observed activity at a vertex, the reconstructed EMEG signal at that vertex for each stimulus word (numbered 1 to *n*) is correlated (dark blue arrows) with the output of some model (here loudness) for the same word. This set of matched correlations is evaluated against a set of mismatch correlations, generated by correlating the same EMEG signals with the predicted output for five randomly selected different words (unfilled arrows). Time is from stimulus onset. **(B)** Illustration of the underlying matched and mismatched distributions when the instantaneous F0 model is tested at a source in LH auditory cortex at 95 ms, together with the means and quartiles of these distributions.

The procedure we describe here is a form of cross-correlation analysis conducted in a whole-brain searchlight context, across a large array of cortical regions (sources), and over multi-second time periods sampled at millisecond intervals. To meet the requirements for the analysis procedure in this context—that it match the predictions of specific models against brain-wide neural activity at different time-lags between system input and neural output—we adopt a two stage process, as laid out in Figures 2, 3A.

We illustrate this process using the model for instantaneous loudness-sones, as specified in Section “The Models for Instantaneous Loudness” above. This model is applied to a set of 480 words for which EMEG data have been collected and where distributed source estimation techniques can estimate the distribution of this neural activity, source by source, across a triangularized mesh of the cortical surface (see Section “Source Reconstruction”). The EMEG signals at each source/vertex and the outputs of the model for each word are imported into Matlab.

At the first stage (see Figure 2A), for some time-lag of interest, *l*, for each vertex, *v*, two sets of pairings are created, one “matched” and one “mismatched.” The matched set pairs the neural activity recorded from *v* over the course of a given word with the output signal generated for the same word by the model of interest (in this case the full loudness-sones contour, predicting the neural representation of the speech envelope). This is repeated for all 480 words in the sample, and delivers an empirical distribution of similarities at each vertex (measured using Pearson's ρ). The mismatched set pairs the same EMEG recording at *v* with the output produced by a different word, randomly selected using Matlab's rand() function. In the analyses reported here we computed mismatch similarities for five randomly selected different words, yielding 2400 pairings (see Figure 3A).

These matched and mismatched distributions were normalized using the Fisher-Z transform, and then evaluated for similarity using the one tailed two-sample *t*-test (not assuming equal variances), between the matched and mismatched. If the resulting *p*-values were less than the previously agreed alpha (see Section “Statistical Considerations” below), these results were mapped back onto the cortical surface. These matching procedures were repeated at every vertex, for a given value of time-lag, *l*. This generates a brain-wide statistical parametric map (SPM), picking out those vertices where the population of matched correlations differs from the null distribution of mismatched correlations.

In the second stage of the analysis process, the above procedure was repeated at 5ms intervals (Figure 2B) across a range of time-lags (−200 < *l* < 700 ms), covering the range of plausible latencies (0–700 ms) and a short, pre-stimulation range (−200 to 0 ms) during which we would expect to see no significant match. This produces an SPM which changes over lag, as the lag is varied, revealing the evolution of match and mismatch for a given model's predicted behavior with observed behavior over cortical space and time. We refer to the observation of significant matches at a specific lag as “model expression.”

This set of procedures was followed for the models described in Sections “The Models for Instantaneous Loudness” and “The Model for Instantaneous F0”. The statistical assumptions underpinning these procedures are outlined below.

#### Statistical considerations

By its nature, electrophysiological data has a low signal-to-noise ratio (SNR). The standard way of improving SNR is data-averaging, typically over different stimuli over different subjects. However, in the present study we want to check for the presence of stimulus-specific signal components, which rules out averaging over dissimilar stimuli (i.e., different words). A further methodological challenge is that SNR is likely to vary from vertex to vertex in the source space. We have chosen therefore to perform meta-analyses of signal detection statistics at each vertex and time lag.

The cross-correlation function is the primary tool we use for detection of a specific signal *s*(*t*) as a sub-component of unknown latency *l* in a measured activity *a*(*t*). This function evaluates the lagged correlation of *s* with *a* with a lag of *l*. The mean-corrected cross-correlation function indicates the strength of the agreement between signal and activity, ignoring differences in mean level. The cross-correlation metric allows for differences in the variance of *s* and *a* across different stimuli. It is linked to an implicit regression model where *a*(*t* + *l*) = β*s*(*t*) + *n*(*t* + *l*), where *n* denotes unexplained noise.

A key consideration is that there may be hidden commonalities amongst both the signals predicted by the candidate models and the electro-physiological activity across the stimulus set, which would tend to make the mean value of the cross-correlation function *c*(*s*_*i*_, *a*_*i*_, *l*) greater than 0 (we indicate the specific input stimulus by the subscript *i*). We can characterize these commonalities using two elaborated regression models: first of *s*_*i*_ = *s*′_*i*_ + γ_*i*_ω_*s*_, where ω_*s*_ represents the commonalities between signals predicted by the candidate models (in the case of the loudness models, for instance, all words rise in loudness at the start of their duration, and lower in loudness toward the end); and second, of *a*_*i*_ = *a*′_*i*_ + μ_*i*_ω_*a*_, where ω_*a*_ represents the commonalities between the electro-physiological signals caused by the region of the cortex reacting to the stimuli set. The presence of ω_*s*_ and ω_*a*_ and any shared variance they may have at each latency induces shared variance and hence a contribution to the cross-correlation of *s*_*i*_ with *a*_*i*_.

We address this issue by comparing the cross-correlation values for matched signal and activity *c*(*s*_*i*_, *a*_*i*_, *l*) with the values obtained when the signal and activity are mismatched *c*(*s*_*i*_, *a*_*j*_, *l*) where *i* ≠ *j*. Our local null hypothesis is that when *s* is present in *a* at lag *l* then the mean matched cross-correlation 𝔼 [*c*(*s*_*i*_, *a*_*i*_, *l*)] will be greater than the mean mismatched cross-correlation 𝔼[*c*(*s*_*i*_, *a*_*j*_, *l*)]. We compared 480 matched cross-correlations with 2400 mismatched cross-correlations by means of a two sample *t*-test for the matched sample using the mean of the mismatched sample as reference. For each time lag we have a statistical parametric map (SPM) of one-tailed *p*-values of these *t*-tests at 2562 vertices per hemisphere, in source space. The procedure then is to report those vertices (if any) which have a *p*-value lower than the specified threshold α = 0.05 (or as otherwise set), where the peak of these values is assumed to reflect the primary neural realization of the output of the model under investigation.

The convention in brain imaging studies is to set the specified threshold α for single hypothesis tests at *p* ~ 2.3 × 10^−2^, the equivalent of 2 standard deviations (2σ) from the mean of the null-distribution (assuming the distribution's normality). In this paper, we decrease α to the equivalent of 5σ (*p* ~ 2.9 × 10^−7^), to ensure that the evidence we present for the expression of particular models is unequivocally robust. Furthermore, when testing for effects across a brain volume (here indexed by vertex *v* and lag *l*) without a specific regional hypothesis in mind, allowance must be made for multiple testing. While we can directly specify false alarm rates α_*vl*_ for each simple null hypothesis *H*_*vl*_, we are mainly interested in the resulting family-wise false alarm rate for the multiple null hypothesis ∪*H*_*vl*_. Where there is statistical dependence between the various tests it may be difficult to calculate the exact value of this rate. A variety of approximation techniques, of which the most familiar is the “Bonferroni correction, ” have been developed to estimate the rate's upper bound.

Calculating the family-wise false alarm rate so that it accurately reflects what we know about the data being tested can be difficult. In the current study, some of the data used in the tests will be dependent on others (because of spatial and temporal similarities between neighboring vertices and lags). However, it is very difficult, if not impossible, to get accurate estimations of, for instance, the spatial dependencies between sources. In the present study, rather than accept assumptions about the dependencies that are hard to justify, we assume the data at each vertex and lag are independent (a “worst case” scenario). As a result, the reader should be aware that the type II error rate is likely to be high, making the results “conservative.”

Assuming independence between each of the tests, we can use an exact formula for the family-wise false alarm rate, 1 − ∏_*v*, *l*_ (1 − α_*vl*_). When all the α_*vl*_ have the same value α we get the specified multiple hypothesis test false alarm rate α by choosing $\alpha^{*}=1-\sqrt[{N}]{1-\alpha }$ where *N* is the combined number of vertices and lags tested (927,444 in this study). The “corrected” α, α^*^, will therefore be a *p*-value of approximately 3 × 10^−13^. *p*-Values greater than this value will not be deemed significant.

### MEG and EEG methods and materials

#### Participants

Twenty right-handed native speakers of British English (13 men, mean age = 25 years, range = 19–36) were recruited. All gave informed consent and were paid for their participation. The study was approved by the Peterborough and Fenland Ethical Committee (UK).

***Stimuli***. The study used 480 English verbs (e.g., *talk*, *follow*) or simple verb phrases (e.g., *I walk*), half of which had past tense inflections (e.g., *arrived*, *jumped*). These materials were prepared for another experiment, and it is assumed that their linguistic properties were independent of the basic auditory parameters being tested for here. The stimuli were recorded in a sound-attenuated room by a female native speaker of British English onto a DAT recorder, digitized at a sampling rate of 22 kHz with 16-bit conversion, and stored as separate files using Adobe Audition (Adobe Inc., San Jose, California). They averaged 885 ms in length.

#### Procedure

Each trial began with a central fixation cross presented for an interval jittered between 250 and 400 ms. The cross remained until the completion of the spoken words, followed by a blank screen for 1400 ms. For the majority of trials the participants simply listened to the stimuli. For 8% of the trials, a one-back memory task was introduced to encourage the participants to listen attentively. On these trials, a written word or phrase was presented after the blank screen, and the participant indicated whether this matched the preceding spoken word. Presentation of stimuli was controlled using Eprime software (Psychology Software Tools, Inc., Sharpsburg, Pennsylvania). The (monophonic) stimuli were presented binaurally at approximately 65 dB SPL via Etymotics earpieces. Each item was presented twice in pseudorandom order, split into eight blocks 6 min long. Each participant received 10 practice trials, including four one-back memory trials.

#### EMEG recording

Continuous MEG data were recorded using a 306 channel VectorView system (Elektra-Neuromag, Helsinki, Finland) containing 102 identical sensor triplets (two orthogonal planar gradiometers and one magnetometer) in a hemispherical array situated in a light magnetically-shielded room. The position of the head relative to the sensor array was monitored continuously by four Head-Position Indicator (HPI) coils attached to the scalp. Simultaneous EEG was recorded from 70 Ag–AgCl electrodes placed in an elastic cap (EASYCAP GmbH, Herrsching-Breitbrunn, Germany) according to the 10/20 system, using a nose electrode as reference. Vertical and horizontal EOG were also recorded. All data were sampled at 1 kHz with a band-pass filter from 0.03 to 330 Hz. A 3-D digitizer (Fastrak Polhemus Inc., Colchester, VA) recorded the locations of the EEG electrodes, the HPI coils and approximately 50–100 “headpoints” along the scalp, relative to three anatomical fiducials (the nasion and left and right pre-auricular points).

#### Data pre-processing

Static MEG bad channels were detected and excluded from subsequent analyses (MaxFilter version 2, Elektra-Neuromag, Stockholm, Sweden). Compensation for head movements (measured by HPI coils every 200 ms) and a temporal extension of the signal–space separation technique (Taulu et al., 2005) were applied to the MEG data. Static EEG bad channels were visually detected and removed from the analysis (MNE version 2.7. Martinos Center for Biomedical Imaging, Boston, Massachusetts). Artifact components associated with eye-blinks and saccades were automatically detected and projected out using the independent component analysis tools of EEGLAB (Delorme and Makeig, 2004). The EEG data were re-referenced to the average over all channels. The continuous data were low-pass filtered to 55 Hz (zero-phase shift, overlap-add, FIR filtering) and epoched with respect to stimulus onset. Epochs of 2300 ms were used: 1300 ms (the length of the longest word) from stimulus onset, plus 200 ms before and 700 ms after. Epochs in which the EEG or EOG exceeded 200 μV, or the value on any gradiometer channel exceeded 2000 fT/m were rejected from both EEG and MEG datasets. Epochs for each participant were averaged over both stimulus repetitions.

#### Source reconstruction

The location of the cortical current sources was estimated using MNE (Hämäläinen and Ilmoniemi, 1994), neuro-anatomically constrained by MRI images obtained using a GRAPPA 3D MPRAGE sequence (TR = 2250 ms; TE = 2.99 ms; flip-angle = 9°; acceleration factor = 2) on a 3T Tim Trio (Siemens, Erlangen, Germany) with 1 mm isotropic voxels. For each participant a representation of their cerebral cortex was constructed using FreeSurfer (Freesurfer 4.3, Martinos Center for Biomedical Imaging, Boston, Massachusetts). The forward model was calculated with a three-layer Boundary Element Model using the outer surface of the scalp and the outer and inner surfaces of the skull identified in the structural MRI. Anatomically-constrained source activation reconstructions at the cortical surface were created by combining MRI, MEG, and EEG data. The MNE representations were downsampled to 2562 vertices per hemisphere, roughly 6 mm apart, to improve computational efficiency. Representations of individual subjects were aligned using a spherical morphing technique (Fischl et al., 1999). Source activations for each trial were averaged over participants, resulting in an “average participant” current estimation on which the analysis was carried out. We employed a loose-orientation constraint (0.2) to improve the spatial accuracy of localization. Sensitivity to neural sources was improved by calculating a noise covariance matrix based on the 200 ms prestimulus period. We assume, in this study, that the outputs of the models are encoded in the positive component of the cortical current; with this in mind, the resulting current estimation was half-wave rectified. Reflecting the reduced sensitivity of MEG sensors for deeper cortical activity (Hauk et al., 2011), sources located on the cortical medial wall and in subcortical regions were not included in the analyses reported here.

#### Visualization

**Figure 4:**
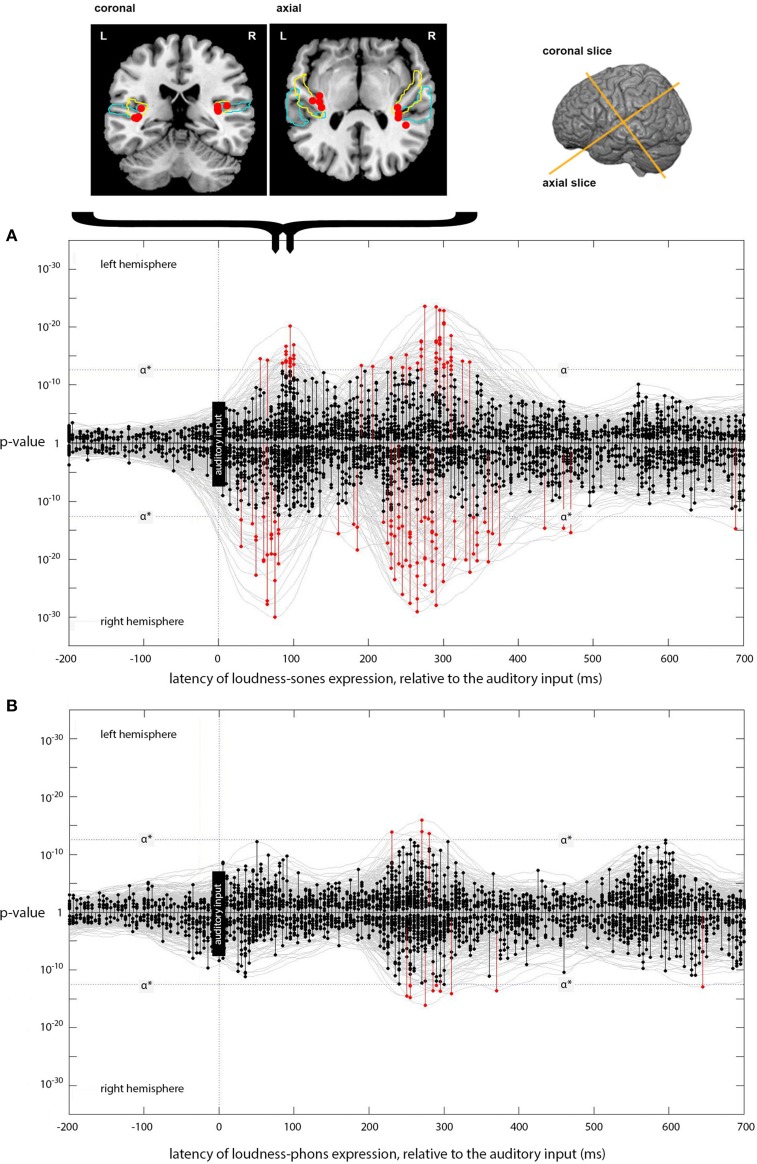
**Expression of loudness-sones and loudness-phons models at different lags. (A)** Plots the expression for the instantaneous loudness-sones model (generating a predicted neural speech envelope) across processing lags from −200 to +700 ms, relative to the auditory input. Results for the left and right hemispheres separately are plotted mirror-wise across the mid-line. Vertices which match the output of the sones model at or above the stipulated α^*^ (*p* ~ 3 × 10^−13^) are plotted in red. The cortical locations of the most significant four vertices in the initial RH peak at 75 ms and the corresponding LH peak at 90 ms are indicated on the coronal and axial slices above **(A)**. Probabilistic anatomical landmarks are provided for planum temporale (light blue) and Heschl's gyrus (yellow) (Rademacher et al., 1992; Morosan et al., 2001; Fischl et al., 2004). **(B)** Shows the parallel results, plotted in the same way, for the expression of the instantaneous loudness-phons model.

The cortical slices in Figures 4, **6**. use the visualization software MRIcron (Georgia State Center for Advanced Brain Imaging, Atlanta, Georgia) with results mapped to the high-resolution colin27 brain (Schmahmann et al., 2000). For labeling purposes, two anatomical regions (planum temporale and Heschl's gyrus) were mapped onto the figure using probabilistic atlases (Rademacher et al., 1992; Morosan et al., 2001; Fischl et al., 2004).

## Results

### Instantaneous loudness-sones and instantaneous loudness-phons

The model-testing procedure described above was first used to determine whether the neural signal corresponding to the output of the instantaneous loudness-sones model could be identified in a brain-wide spatiotemporal search of EMEG source data for a large set of naturally spoken words. The results for this psychologically plausible measure of the amplitude envelope of the speech were then compared with those produced with a less plausible measure (instantaneous loudness-phons), which does not capture the channel-specific compression imposed on the stimulus by the neurophysiological properties of the human auditory system (Glasberg and Moore, 2002). Figure 4 shows the temporal distribution of those vertices, across processing lags from −200 to +700 ms relative to the incoming auditory input, where the statistical evidence for a match between the reconstructed EMEG data and the signal computed using the loudness-sones and loudness-phons models exceeds the stipulated α^*^ level.

Focusing on the separate left and right hemisphere (LH and RH) plots for loudness-sones (Figure 4A), we see an initial cluster of significant effects peaking at a lag of around 75 ms in the RH and at 90 ms in the LH. In each case, there is some evidence for earlier expression of the sones model, starting at 55 ms in the LH and 30 ms in the RH. The peak vertices for the main clusters at 75 and 90 ms both fall in auditory cortex (Heschl's gyrus). The cluster coordinates are (−35, −28, 10) and (34, −28, 14) for LH and RH respectively. The loudness-phons model shows no significant vertices in this latency range (Figure 4B).

We compared the fits of the loudness-sones and loudness-phons models using the *p*-values that the analysis procedure returns as a summary statistic. To this end we perform a Wilcoxon signed rank, paired, one-sided test, that the average *p*-value over all sources for loudness-sones is less than loudness-phons (*z*_stat_ = − 12, *p* < 0.01, *n*_1_ and *n*_2_ = 5124). The loudness-phons model is, on average, a weaker fit to the reconstructed source current (Figure 4B), despite the fact that the sones and phons models generate contours that are highly correlated (70% shared variance).

**Figure 5:**
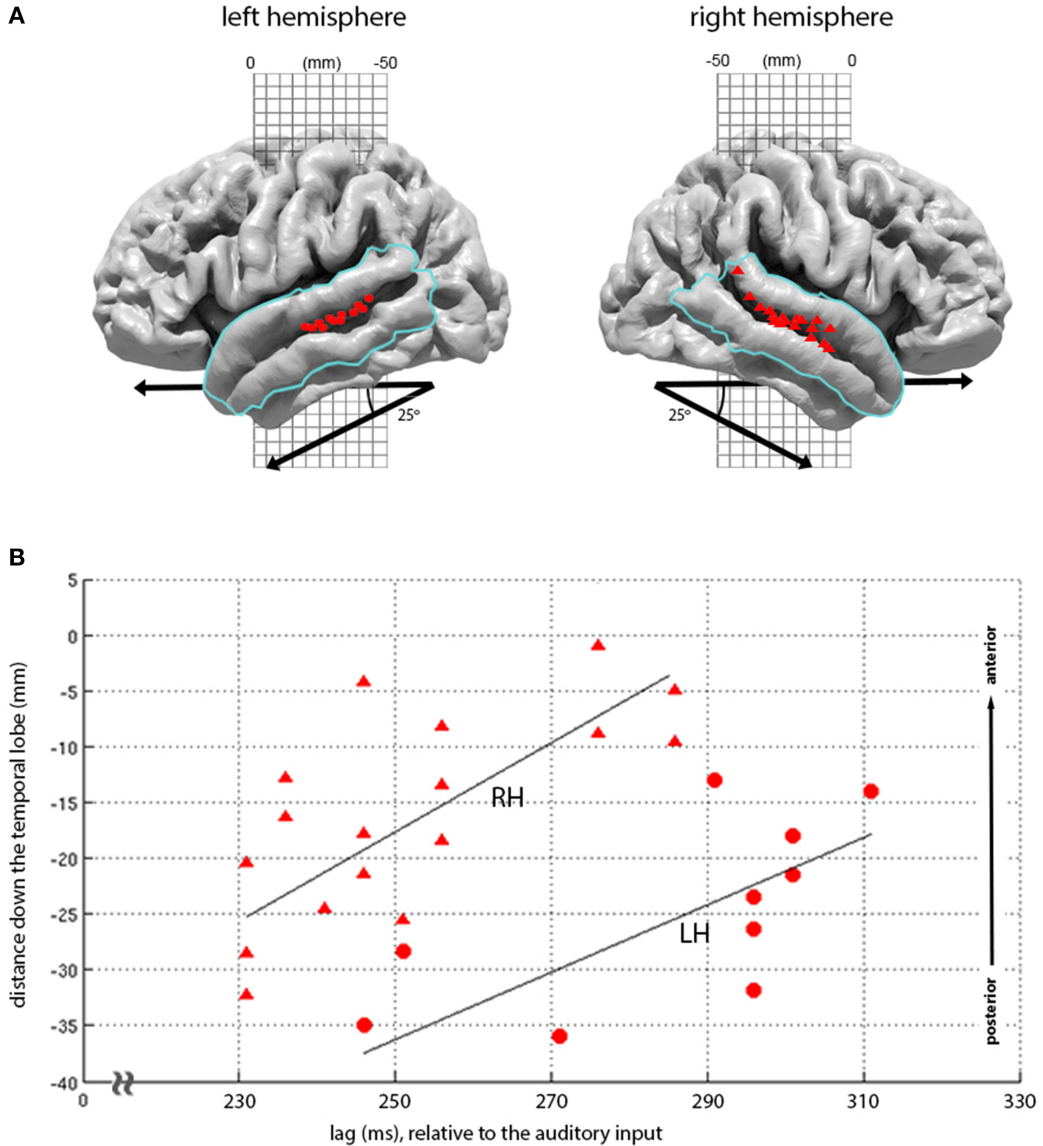
**Topography of loudness-sones expression at 210-330 ms lags. (A)** Plots the second major cluster of instantaneous loudness-sones expressions rendered onto the LH and RH cortical surface, overlaid with a mask (blue line) covering the superior and middle temporal gyrus). The locations for all vertices displaying significant matches in this mask, for the time period of 210 and 330 ms relative to the auditory input, are displayed as red circles (LH) and red triangles (RH). **(B)** Plots the same vertex matches, as a joint function of the distance down the temporal lobe (measured down a 25° diagonal off the MNI-space *y* axis) against the lag of each vertex match. A least-squares linear regression (with the line of best-fit plotted in black) demonstrates a linear relationship between lag and the location of significant vertices (LH, RH: ρ = 0.70, 0.70, *p* = 0.01, 0.001).

The loudness-sones model is also expressed at lags beyond the initial 70–95 ms period. Figure 4A shows a substantial second cluster of significant vertices at lags extending from approximately 210 to 330 ms in both LH and RH, with a somewhat larger and denser cluster on the right. The peak vertices for these later clusters of matching vertices (see Figure 5A) no longer fall in auditory cortex; rather, they are distributed along the superior temporal sulcus (STS) between the superior and middle temporal gyri (STG/MTG). A secondary cluster of vertices, expressed over the same time periods, is located along the dorsal edge of the Sylvian fissure, superior to HG and the insula.

The later expression of loudness-sones along the STS follows a marked pattern, moving down the STS as a function of lag, with the earlier effects seen more posteriorly and dorsally and the later effects progressing along the STS toward the temporal poles. Figure 5B shows that this distribution of expression over lags and locations is largely parallel in the two hemispheres, with equivalent linear relationships between vertex position and temporal lag (LH ρ = 0.70, *p* = 0.01; RH ρ = 0.70, *p* = 0.001), and with similar slopes (*t*_stat_ = 0.70, *p* = 0.5, *n* = 26). However, as with the primary peaks at 75–90 ms, these effects are earlier and stronger in the RH, with model expression appearing some 20 ms earlier, involving 17 rather than 10 significant vertices, and extending further down the STS.

### Instantaneous F0

The instantaneous F0 contour of speech plays an important role in the prosodic interpretation of words and utterances. Unlike loudness, however, F0 does not reflect variations in the magnitude of a given sound, but rather variations in the fundamental frequency (the glottal pulse rate) at which voiced sounds are being produced. Here we ask whether we can identify the spatial locations and processing lags at which the model for F0 is being expressed.

**Figure 6:**
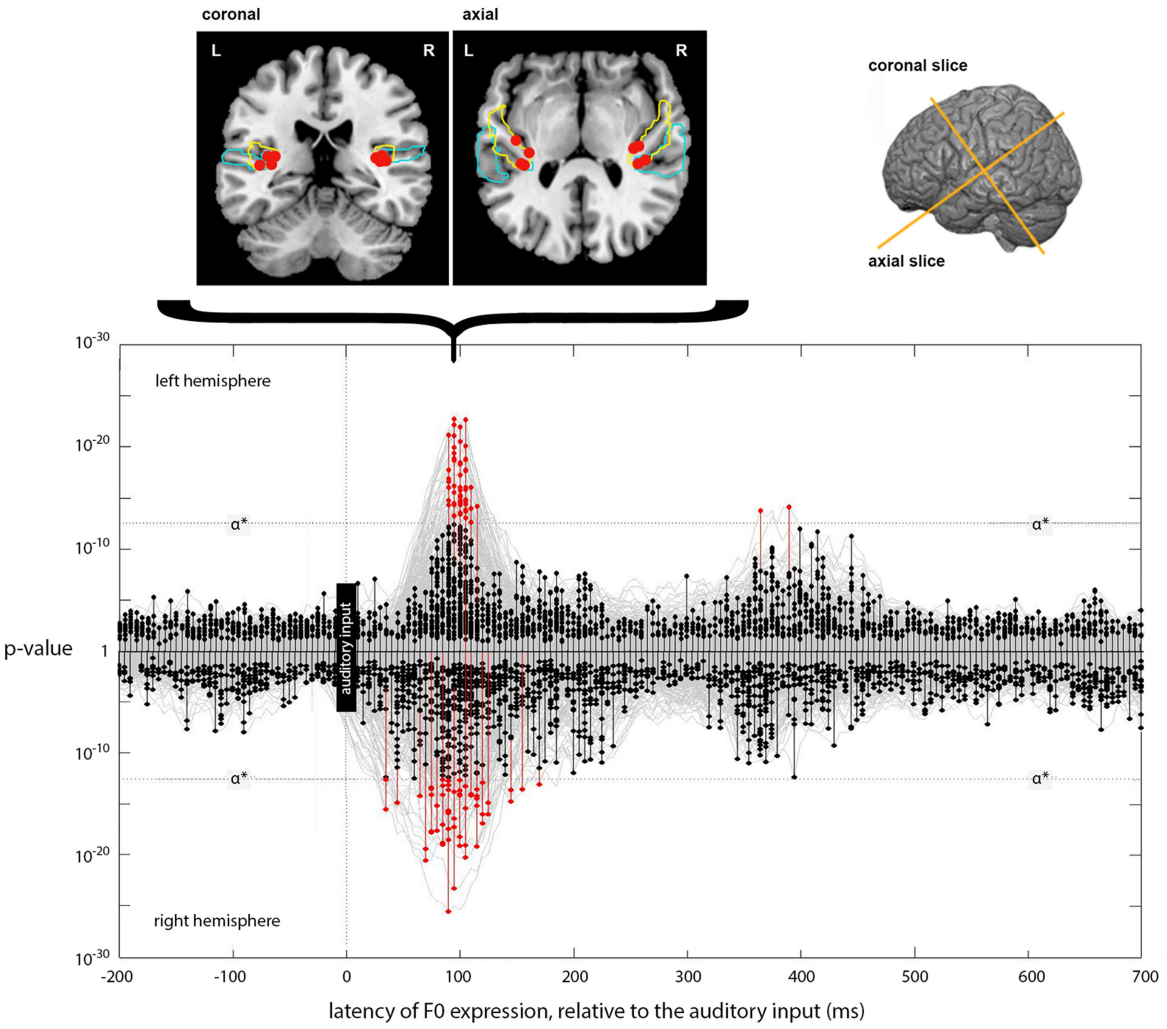
**Expression of the instantaneous F0 model across lags**. The main panel plots the expression of the instantaneous F0 model (generating a predicted F0 contour) across processing lags from −200 to +700 ms, relative to the auditory input. Results for left and right hemispheres separately are plotted mirror-wise across the mid-line. Vertices which match the output of the F0 model at or above the stipulated α^*^ (*p* ~ 3 × 10^−13^) are plotted in red. The positions of the most significant four vertices at 90 ms, for both LH and RH, are plotted on the cortical slices above the main panel. These locations are bilateral and symmetric, and are focused around Heschl's Gyrus (colored blue). The MNI-space coordinates for the most significant match in the left and right hemispheres are (−37, −26, 9) and (35, −27, 13), respectively. Later expression also occurs in the left hemisphere at a lag of 365 and 390 ms; located at (−41, −24, 22) and (−40, −25, 22), respectively.

The results (Figure 6) match those for loudness-sones in two major respects. First, there is a strong, bilateral expression of the F0 model, with peak vertices falling at a lag of 90 ms in both LH and RH. Second, the cluster locations for these peak vertices are appropriately located in auditory cortex, in and around Heschl's Gyrus, with MNI cluster coordinates of (−37, −26, 9) and (35, −27, 14) for LH and RH, respectively.

Unlike the expression for loudness-sones, there is only weak evidence for a later phase of neural computation that requires further entrainment of the F0 contour. Although there are two LH vertices showing significant expression at lags of 365 and 390 ms, located on the dorsal edge of the Sylvian fissure above HG at (−41, −24, 22) and (−40, −25, 22), respectively, this is not comparable to the loudness-sones results either in extent or in the type of structured patterning over time that we see between 210 and 330 ms in the neural expression of the loudness-sones model.

## Discussion

The application of the model testing procedure to the analysis of the electrophysiological activity of the brain is based on the assumption that neural activity can be characterized in terms of models that are expressed in different brain regions and at different time-lags, as a given input is being analyzed and interpreted. Here we tested a set of well-established models drawn from auditory neuroscience. We applied these on a brain-wide searchlight basis to the reconstructed neural activity elicited by a large set of naturally spoken words.

For two of these models—instantaneous F0 and instantaneous loudness-sones—we found strong evidence for their expression in auditory cortex at plausible time-lags (Griffiths et al., 2010; Kubanek et al., 2013), with extensive further expression of the loudness-sones model at later time lags in temporal cortex, moving from more posterior to more anterior locations over time (~250–400 ms). The third model, instantaneous loudness-phons, which is psychophysiologically unrealistic, generated a significantly poorer and statistically marginal fit to the brain data.

As with most hypotheses concerning computation in cortex, these specific loudness-sones and F0 models only approximate the mechanisms transforming the speech signal to dendritic current in auditory cortex. The relative evidence of two competing models can be tested in two ways: testing both to see which has higher evidence of entrainment in a source, or looking for evidence from other studies that supports or falsifies the models under consideration. For instance, knowledge that the cochlea uses frequency bands increases the plausibility of a model that includes reference to these bands over a model that does not, even if the outputs of the models are highly correlated and the evidence of cortical entrainment at a source is equivalent.

The difference between the sones and phones fits, established by the Wilcoxon test, is an example of this first case, indicating that the loudness-sones model is a more plausible hypothesis than the loudness-phons model with respect to how the speech envelope is encoded in auditory cortex (presumably because the loudness-sones transformation includes channel specific compression like that observed in the cochlea). Nonetheless, the use of summary statistics can only indicate that loudness-sones is the better model *on average*; there may be individual sources for which loudness-phons is the better model, and we do not test for such instances in this study.

It is worth noting that the mean values of the ρ distributions for the null and alternate hypotheses (examples of which can be seen in Figure 3b) are low for all vertices and lags—almost always less than 0.3. This means the unexplained variance remains relatively high. The reasons for this include: (1) the specific models are only approximations of the true neural mechanisms for F0 and loudness; (2) the outputs of other models in neighboring sources may be “leaking” into those sources carrying loudness and F0 due to inaccuracies in the source reconstruction procedure; (3) as part of the source estimation procedure, we have assumed that there are 2562 vertices per hemisphere. This assumption is almost certainly an underestimation, which would lead to inaccuracies in the reconstruction; (4) source reconstruction through MNE does not guarantee error-free localization due to the inverse problem (Hauk et al., 2011).

### Instantaneous loudness-sones and loudness-phons

The epoch over which we performed our instantaneous loudness analyses corresponds to the speech envelope of the word. This is hypothesized to be functionally critical in speech comprehension (e.g., Boemio et al., 2005; Ghitza and Greenberg, 2009; Peelle and Davis, 2012), with recent studies highlighting the role of the speech envelope as a driver of theta-band (3–8 Hz) slow-wave oscillations in neural speech analysis (Ghitza, 2012; Giraud and Poeppel, 2012). This rhythmic variation is correlated to the syllabic structure of the speech input, and is claimed to play a critical role in modulating and integrating higher frequency gamma-band analysis of the spectro-temporal fine structure of speech. Within the “asymmetric sampling in time” approach (Poeppel, 2003; Giraud et al., 2007), this emphasis on the dynamic processing role of the speech envelope is associated with claims for strong hemispheric specialization (Boemio et al., 2005; Abrams et al., 2008), where the right hemisphere is primarily sensitive to slow-wave variation related to syllable structure, while the left hemisphere is biased toward gamma-band analyses of temporally fine-grained phonetic detail (Zatorre and Gandour, 2008).

A number of studies have used intra-cranial techniques to correlate the speech envelope with neural activity. Nourski et al. (2009), recording directly from an electrode strip placed along Heschl's gyrus in pre-surgical epilepsy patients, found envelope following for time-compressed speech in core auditory cortex (posteromedial HG) but little or no time-locking in auditory belt areas (anterolateral HG). More recently Kubanek et al. (2013), using an ECOG electrode grid placed over the left hemispheres of five pre-surgical patients, measured responses during attentive listening to a spoken story to determine whether the speech envelope is tracked over wider cortical regions. Using a representation of the speech envelope similar to loudness-phons, they found a significant correlation with neural activity in the high gamma range (75–115 Hz) in belt regions of auditory cortex at a lag of around 90 ms. Only weak correlations, at similar lags, were found for electrodes placed elsewhere in the left hemisphere, including STG. In a third study by Aiken and Picton (2008) the Hilbert envelope of the speech wave (log transformed) was correlated with source localized EEG. Matches were found in anterior auditory cortex for both left and right hemispheres, with lags at around 175 (left) and 180 (right) ms.

Although the lags in Kubanek et al. and Aiken and Picton differ somewhat from those in the current study, these discrepancies may be accounted for by differences in methodology. Both Aiken and Picton (2008) and Kubanek et al. (2013) report the average lag of the highest correlation values found during cross-correlation; in the current study, the latency is based on the time of the source's minimum *p*-value, over all tested lags. Moreover, Kubanek et al. examined the 75–115 Hz range, whereas Aiken and Picton examined 0.15 and 50 Hz, and we examined 0.15 and 55 Hz.

Relating these results to those from studies that estimate impulse response functions (IRFs) for envelope-based models is more difficult. An IRF estimated from the loudness-sones model that takes the form of two single unit impulses present at lags of 75 ms in the RH and at 90 ms in the LH would be equivalent to the findings of this study, but in general, the envelope-based models tested with IRF-estimation techniques are different to those used here (e.g., log-RMS of the signal in Lalor et al., 2009 and Power et al., 2012; the spectrogram model of Yang et al., 1992 in Ding and Simon, 2012) and the estimated IRFs are more complex than single unit impulses (e.g., Aiken and Picton, 2008; Ding and Simon, 2012, 2013; Zion Golumbic et al., 2013).

Although the models/IRFs of IRF-estimation studies and the models considered here mean the hypotheses tested are not directly equivalent, comparison between the studies regarding the lag of speech envelope information (relative to stimulus exposure) is still possible. Any IRF that shows a non-zero value or values at a positive lag is evidence that loudness information is affecting the behavior of the underlying source current at that (those) lag(s). Thus the finding that the behavior of regions of the cortex are composed from the output of an envelope model convolved with an IRF peaking (or dipping) at (for instance) a 50 ms lag (Ding and Simon, 2013; Zion Golumbic et al., 2013), implies that these regions are carrying information based on the envelope of the speech stream at this lag. It is not clear how this information relates to the convolved entrainment of the speech envelope found 30ms later in this study (and/or the lags found in Aiken and Picton, 2008 and Kubanek et al., 2013).

Beyond the initial bilateral expression of loudness-sones in the 70–90 ms range in auditory cortex, loudness-sones shows cortical entrainment at a sustained progression of lags from 210 to 330 ms down the superior temporal sulcus. The progression of speech analysis anteriorly along the superior and middle temporal lobes is consistent both with older evidence for a ventral progression reflecting phonological and lexical analysis of speech (e.g., Scott et al., 2000; Davis and Johnsrude, 2003), and with recent meta-analyses (DeWitt and Rauschecker, 2012) suggesting the sequential emergence of word-based object-centered processes, moving from phoneme-based processes in mid-STG and STS to lexical and phrasal units at more anterior sites. The expression of the loudness-sones model throughout this process may reflect a series of discrete computations at different points along the STS, each of which draws upon or is guided by this neural representation of the speech envelope. However, this observation of “sequential expression” in a single direction along the cortex may have other causes. For instance, it may reflect a form of neural propagation wave [where the measured velocity of the hypothetical wave, 0.3 ± 1 m/s for left STS and 0.4 ± 1m/s for right STS, is consistent with velocities estimated in other neural propagation wave studies (Benucci et al., 2007; Reimer et al., 2011; Sato et al., 2012)].

Finally, this pattern of results argues against a strong division of labor between the hemispheres where short-term and long-term variation in the speech waveform is concerned. Several studies infer such a division on the basis of the relative sensitivity of right and left hemispheres to oscillatory variation at different time-scales, both for non-speech and speech sounds (e.g., Boemio et al., 2005; Luo and Poeppel, 2007; Abrams et al., 2008). Indeed, evidence of the output of the loudness sones model (using a representational similarity method) has previously been reported in in HG/mSTG/aPT by Giordano et al. (2013), but only in the left hemisphere.

While such biases may exist, it is clear from the analysis performed here that the loudness-sones model generating the neural speech envelope is being significantly and sustainedly expressed on the left as well as on the right, which indicates that the speech envelope is maintained in both hemispheres until relatively late in the speech analysis process. Nevertheless, the shorter latencies and greater strength of expression in the right hemisphere mirror similar findings in Howard and Poeppel (2009), who propose that a rightward asymmetry in the processing of energy onsets may be the basis of the lateralization of complex functions such as stimulus-driven orienting of attention (Corbetta and Schulman, 2002) and spatial processing (Zatorre and Penhune, 2001; Hausmann et al., 2005), which are both more prominent in the right hemisphere.

### Instantaneous F0

Although the F0 model exhibits similar entrainment to the loudness-sones model in the early stages of speech processing (insofar as it is expressed in the auditory cortex between 70 and 100 ms), there is no later expression of the F0 model at delays of 210–330 ms. This suggests that the F0 contour of a word may play a qualitatively different role to the speech envelope in the processes underpinning speech perception and word recognition. One implication is that it is not involved to the same extent in the later processing of lexical and phrasal units, as hypothesized for loudness-sones.

Henry and colleagues have previously shown that the frequency of a sound wave is entrained in cortical current (Henry and Obleser, 2012; Henry et al., 2014). It is difficult to know if the Henry et al. studies and the current study are dealing with the same process. The Henry et al. studies test for entrainment of complex tones, slowly modulated in frequency, to EEG field measurements; the F0 model tested in the current study tracks the changing F0 values of syllables. It seems reasonable to assume that the two processes, both tracking frequency changes in cortical current, might be related, but without running the F0 model on the complex tones of Henry et al. it is not clear to what extent.

### Overview

The results presented here confirm that the contours of instantaneous loudness-sones and instantaneous F0 are entrained to the source current in various locations of temporal cortex. They also support the presence of a loudness model that reflects the compression that takes place in the cochlea. The results also indicate that, despite the difficulties of estimating cortical current using EMEG reconstruction techniques (Sharon et al., 2007), this estimation is accurate enough to allow the detection of cortical locations at which entrainment is taking place. This suggests that entrainment located with source-reconstructed current may complement entrainment located with more invasive techniques, such as intracranial-EEG. The results also indicate that the signals are strong enough to survive the requisite multiple correction testing when searching in unmasked source-space, even with the (unlikely) assumption of independence between testing locations and time points.

Instantaneous loudness and instantaneous F0 are not the only features of interest in speech perception; qualities such as degree of periodicity and vowel quality are also important. Moreover, it seems likely that perceptual versions of these instantaneous models (which temporally integrate the instantaneous contour) will isolate activity in other regions of the temporal lobe at different latencies. It remains to be seen how many of these are encoded as the magnitude of electro-physiological current in specific regions.

### Conflict of interest statement

The authors declare that the research was conducted in the absence of any commercial or financial relationships that could be construed as a potential conflict of interest.
